# Analytical expressions for the reconstructed image of a homogeneous cylindrical sample exhibiting a beam hardening artifact in X-ray computed tomography

**DOI:** 10.3233/XST-18378

**Published:** 2018-09-20

**Authors:** Tsukasa Nakano, Yoshito Nakashima

**Affiliations:** National Institute of Advanced Industrial Science and Technology (AIST), Tsukuba, Ibaraki, Japan

**Keywords:** Beam hardening, cupping artifact, exact solution, phantom, polychromatic X-ray spectrum, X-ray CT

## Abstract

**BACKGROUND:**

Cylindrical phantoms are often imaged by X-ray computed tomography (CT) to evaluate the extent of beam hardening (or cupping artifact) resulting from a polychromatic X-ray source.

**OBJECTIVE:**

Our goal was to derive analytical expressions for the reconstructed image of a homogeneous cylindrical phantom exhibiting a cupping artifact, to permit a quantitative comparison with experimental cupping data.

**METHODS:**

A filtered backprojection method was employed to obtain the analytical cupping profile for the phantom, assuming that the projection data could be approximated as a power series with respect to the sample penetration thickness.

**RESULTS:**

The cupping profile was obtained analytically as a series of functions by employing Ramachandran filtering with an infinite Nyquist wavenumber. The quantitative relationship between the power series of the projection and the *n*th moment of the linear attenuation coefficient spectrum of the phantom was also determined. Application of the obtained cupping profile to the evaluation of the practical reconstruction filters with a finite Nyquist wavenumber and to the best choice of the contrast agent was demonstrated.

**CONCLUSIONS:**

The set of exact solutions derived in this work should be applicable to the analysis of cylindrical phantom experiments intended to evaluate CT systems.

## 1 Introduction

Homogeneous cylindrical samples are often used as phantoms in X-ray computed tomography (CT) experiments with a polychromatic X-ray source [1–13, 35]. The resulting CT images containing undesirable cupping artifacts [1–3, 5, 8–11, 14] can be used to assess the extent of beam hardening by the CT system. To do so quantitatively, the theoretical line profile of the CT value along the radial direction of the reconstructed two-dimensional (2-D) cylindrical sample image [1, 2, 4, 5, 8–10, 14, 15] is required to compare with the experimentally obtained line profile. The theoretical line profile contains undesirable errors due to, for example, the imperfect filtering and discretization [4, 10, 37] if the profile is obtained by the numerical image-reconstruction simulation. The ideal beam-hardening assessment without such undesirable errors is possible if the analytical (not numerical) line profile is available. However, to the best of our knowledge, there is no paper on the general solution for the analytical line profile.

In the present theoretical study, we therefore derived analytical expressions for the reconstructed image of a homogeneous cylindrical phantom exhibiting a cupping artifact by assuming the following; (i) that non-linear projection data can be considered as a power series with respect to the sample penetration thickness and (ii) that Ramachandran filtering with an infinite Nyquist wavenumber can be employed. We also examined the relationship between the power series for the projection data and the *n*th moment of the linear attenuation coefficient (LAC) spectrum.

The obtained analytical solutions were applied to the evaluation of the practical reconstruction filters. Numerical simulations reproducing cupping artifacts for a finite Nyquist wavenumber in association with Ramachandran, Shepp, and Chesler filters were performed for a cylindrical specimen containing an aqueous KI solution to confirm the applicability of the analytically-obtained solutions. The analytical solutions were also applied to the choice of the best contrast agent for a cylindrical aqueous phantom. We searched for the atomic number of the heavy element in the aqueous solution/suspension phantom that induced the least cupping artifact.

## 2 Analytical expressions for a cupping artifact

### 2.1 From the projection to the cupping profile

**Fig. 1:**
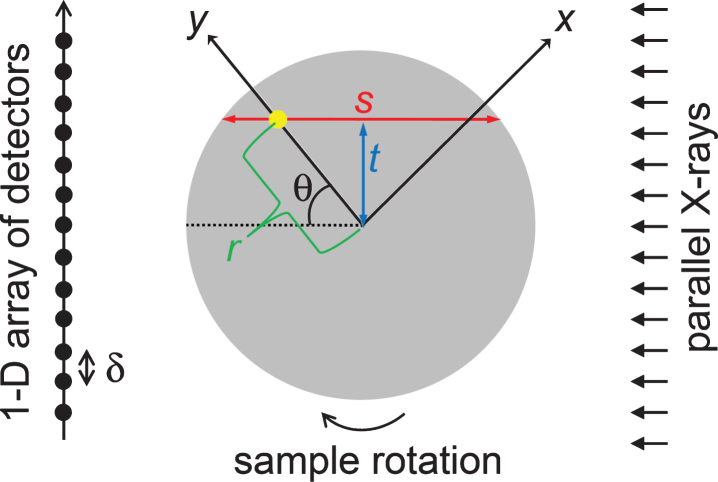
The configuration of a cylindrical sample (in grey) with radius *R* in an imaging system with a detector gap *δ*. The polar coordinates of an arbitrary point (the yellow dot) within the sample are (*r*, *θ*). Another coordinate system (an *x* - *y* system) is rotated by 180 degree during the X-ray emissions. The sample center coincides with the center of rotation.

Herein, we consider the analytical 2-D image reconstruction of a homogeneous cylindrical sample with radius, *R*, using a polychromatic X-ray source and an array of linear detectors (Fig. 1). The bowtie filtration [35] which modulates the original X-ray source spectrum is not considered. The incident parallel X-rays have an initial intensity, *I*(0), and penetrate the sample to a depth of *s*. The attenuated X-ray intensity, *I*(*s*), is measured physically [36] by detectors placed at a constant distance of *δ*. The projection, *p*(*s*), obtained by the detectors is defined as:

(1) $$p(s)\;=\;\ln\left[\frac{I(0)}{I(s)}\right]\;\;\;\;\;\;\;\;\;\;\;\;\;\;\mathrm{for}\;0\;\leq \;s\;\leq \;2R.$$

The 2-D polar and Cartesian coordinate systems, (*r*, *θ*) and (*x*, *y*), respectively, are indicated in Fig. 1. The system is axisymmetric because the sample is homogeneous and cylindrical. Thus, 2-D image reconstruction by CT imagery is essentially identical to deriving the 1-D cupping profile, *f* (*r*), rather than *f* (*x*, *y*) where *f* is the reconstructed value of the LAC obtained by the CT system.

**Fig. 2:**
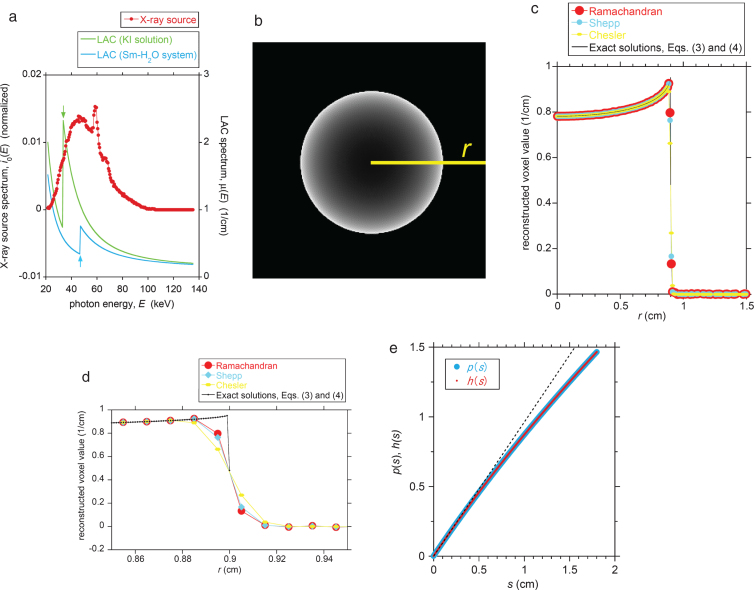
A comparison between the reconstructed voxel values obtained from the analytical expression and a simulated CT image with respect to a homogeneous cylindrical phantom (a 440 mM aqueous KI solution). (a) The normalized photon-energy spectrum of the X-ray source (at an acceleration voltage of 100 kV). Two LAC spectra of the KI solution and of a 160 mM samarium aqueous solution/suspension are superimposed. The K absorption edges of iodine and samarium are indicated by arrows (33 and 47 keV, respectively). (b) The reconstructed CT image of the cylindrical KI solution phantom obtained using the Ramachandran filter with a finite Nyquist wavenumber. The image dimensions were trimmed to 300^2^ voxels (= 3^2^ cm^2^), and the radius, *R*, of the phantom is 90 voxels (= 0.9 cm). The yellow line (the *r*-axis) expanding from the sample center to the image edge is the baseline for the line profile. (c) Line profiles of the reconstructed voxel values along the yellow line in (b) for three reconstruction filters (Ramachandran, Shepp, and Chesler). The true line profile calculated using the exact solution based on Equations (3) and (4) is also plotted for *N* = 100. (d) An expanded view of (c) around the sample edge at *r* = 0.9 cm. (e) The projection, *p*, as a function of *s* as calculated using Equations (1) and (7). The projection, *h*, approximated as a power series of *s* as calculated using Equation (2) via *μ_*n*_*(0) from Equation (A9) is also shown. The linear component of Equation (2) (i.e., *C_1_* *s*) is superimposed by a dotted line to depict the non-linearity of *p* and *h*.

Typical sample LAC and X-ray source spectra are provided in Fig. 2a. The energy-dependent nature of the spectra in Fig. 2a results in a non-linear projection, *p*(*s*), and non-uniform reconstructed value, *f*(*r*), within the phantom. With regard to the non-linearity of the projection, we assume that *p*(*s*) can be suitably approximated by *h*(*s*), which is a power series with respect to the penetration thickness, *s*, written as [1]:

(2) $$h(s)\;=\;\sum_{n=1}^{N}C_{n}s^{n}\;\;\;\;\;\;\;\;\;\;\;\;\;\mathrm{for}\;0\;\leq \;s\;\leq \;2R,$$

where *C_*n*_* is a coefficient and *N* is an integer representing the upper limit of the summation. The energy-dependent LAC spectrum and polychromatic X-ray source spectrum yield the cupping artifact. The physical information of the two spectra is combined and embedded in Equation (2). Thus, it is possible to derive the analytical expressions of the cupping profile by the conventional image-reconstruction algorithm using the non-linear projection data of Equation (2).

Employing the conventional filtered backprojection method [4, 16, 17], we derived an analytical expression for the cupping profile, *f*(*r*), as a series of functions, written as:

(3) $$f(r)\;=\;\sum_{n=1}^{N}{F_{n}\left(R^{2}-r^{2}\right)}^{\frac{n-1}{2}}\;\;\;\;\;\;\;\;\;\mathrm{for}\;0\;\leq \;r\;<\;R$$

and

(4) $$f(R)\;=\;\frac{C_{1}}{2}.$$

Here, the coefficient *F_*n*_* is given by:

(5) $$F_{n}\;=\;\frac{2^{n}}{\sqrt{\pi }}\frac{\Gamma \left(\frac{n}{2}+1\right)}{\Gamma \left(\frac{n+1}{2}\right)}C_{n},$$

where *Γ* is the gamma function. The detailed derivation of Equations (3) and (4) from Equation (2) is described in Appendix 1. It should be noted that Ramachandran filtering with an infinite Nyquist wavenumber (i.e., infinitesimal *δ*) was employed in this derivation.

Equation (3) shows that the linearity (or non-linearity) of the projection is transferred to the constant (or *r*-dependent) distribution of *f*(*r*) as follows. (i) The coefficient for the linear component in Equation (2), *C_1_*, is embedded as *F_1_* in the right-hand side of Equation (3), which contributes to the constant component of *f*(*r*) without cupping artifact. (ii) Each coefficient *C_*n*_* for *n*≥2 corresponding to the non-linear term in Equation (2) occurs in *F_*n*_* on the right-hand side of Equation (3) as the *r*-dependent component of *f*(*r*).

### 2.2 From the spectral moment to the projection

The non-linearity of the projection in Equation (2) is a consequence of the energy-dependence of the LAC spectrum and the broadness of the X-ray source spectrum. We therefore developed a quantitative relationship between the projection and spectra by introducing a spectral moment. The incident X-ray intensity, *I*(0), in Equation (1) can be considered as the sum of all components having various photon energy values, *E*, and thus can be summarized as:

(6) $$I(0)\;=\;\int_{0}^{\infty }i_{0}\left(E\right)\mathrm{dE},$$

where *i*_0_(*E*) is the energy-dependent spectrum of the X-ray source. Hereafter, we assume that *I*(0) is normalized to a value of unity. Following the penetration of the cylindrical sample in Fig. 1 by the X-ray beam, the X-ray intensity is attenuated by Beer’s law to [18]:

(7) $$I(s)\;=\;\int_{0}^{\infty }i_{0}\left(E\right)\exp\left(-\mu (E)s\right)\mathrm{dE},$$

where *μ*(*E*) is the energy-dependent LAC spectrum of the cylindrical sample.

In order to relate the power-low non-linearity in Equation (2) to the exponential non-linearity in Equation (7), we introduce the *n*th moment of the LAC spectrum, weighted by the attenuation due to the sample penetration, defined as:

(8) $$M_{n}(s)\;=\;\int_{0}^{\infty }i_{0}\left(E\right)\mu (E{)}^{n}\exp\left(-\mu (E)s\right)\mathrm{dE}\;\;\;\;\;\;\;\;\;\;\mathrm{for}\;n\;=\;0,\;1,\;2,\;\ldots ,\;N,$$

where *M*_*n*_ (*s*) is the *n*th moment. *M*_*n*_ (*s*) can be normalized by *I*(*s*) as:

(9) $$\mu_{n}(s)=\frac{M_{n}(s)}{I(s)}\;\;\;\;\;\;\;\;\;\;\mathrm{for}\;n\;=\;0,\;1,\;2,\;\ldots ,\;N.$$

Through the process described in Appendix 2, *μ_n_* (0) can be related to *C*_*n*_ as the following recurrence relation:

(10) $$C_{n+1}\;=\;-\nu_{n+1}-\sum_{m=1}^{n}\nu_{n-m+1}\frac{m}{n+1}C_{m}\;\;\;\;\;\;\;\;\mathrm{for}\;n\;=\;0,\;1,\;2,\;\ldots ,\;N-1,$$

where the coefficient *ν_*n*_* is defined by:

(11) $$\nu_{n}\;=\;\frac{{\left(-1\right)}^{n}\mu_{n}(0)}{n!}\;=\;\frac{{\left(-1\right)}^{n}}{n!}\int_{0}^{\infty }i_{0}\left(E\right)\mu (E{)}^{n}\mathrm{dE}\;\;\;\;\;\;\;\;\mathrm{for}\;n\;=\;0,\;1,\;2,\;\ldots N.$$

By the above process, we obtained the analytical relationships among the normalized spectral moment (*μ_*n*_*(0) in Equation (A9)), the coefficient for the non-linear projection (*C_*n*_* in Equation (2)), and the coefficient for the cupping profile (*F_*n*_* in Equation (3)). In the following sections, these exact solutions were applied to the comparison with the practical reconstruction filters and to the search for the best contrast agent in terms of the reduction of the cupping artifact.

## 3 Application of the exact solutions

### 3.1 Effects of reconstruction filters

The analytical expressions derived in Sec. 2 assume Ramachandran filtering with an infinite Nyquist wavenumber. This implies that the detector interval, *δ*, is zero. In practical CT imagery, however, the quantity *δ* is not zero, and Shepp (i.e., sinc) and Chesler (i.e., hanning) reconstruction filters with finite Nyquist wavenumbers are often used in addition to the Ramachandran filter [4, 16]. Thus, conventional numerical simulations to reconstruct a synthetic CT image exhibiting a cupping artifact were performed using such practical filters to demonstrate the applicability of these analytical expressions to practical CT imagery.

The cylindrical sample selected for the reconstruction simulations was composed of a homogeneous aqueous 440 mM KI solution. The radius of the cylinder, *R*, was 0.9 cm and the detector separation distance, *δ*, was 0.01 cm. Thus, the radius of the reconstructed cylindrical sample was (0.9 cm/0.01 cm) = 90 voxels. The LAC spectrum of the sample as calculated using the NIST database [19] is shown in Fig. 2a, in which the X-ray source spectrum at an acceleration voltage of 100 kV [14] is superimposed. The target of the X-ray tube is made of Mo-W alloy. Metal films (Al film of approximately 1 mm and Cu film of 0.1 mm in thickness) are preinstalled on the CTsystem [14].

The 2-D image reconstruction of the cylindrical sample in Fig. 1 was performed with our original computer program. The details of the computer program have been described elsewhere [4, 10, 20]. Briefly, the parallel X-ray beams with the polychromatic X-ray source spectrum are assumed, and the attenuated X-ray intensity is measured on the 1-D array of detectors. The non-linear projection, *p*(*s*), is calculated on the discrete points (separation distance, *δ*) using Equations (1) and (7) for 0≤*s*≤2 *R*. Totally 805 projection data were acquired on the 1-D detector array during the sample rotation of 180°. The original dimensions of the 2-D reconstructed image were 512^2^ voxels, and the obtained sinogram consisted of 512×805 voxels. The convolution backprojection method was applied to the sinogram to reconstruct a 512^2^-voxel image with voxel dimensions of 0.01^2^ cm^2^. Three typical reconstruction filters, the Ramachandran, Shepp, and Chesler filters, with a finite Nyquist wavenumber of 1/(2*δ*) = 50 1/cm, were employed in the computer program. The grey scale of the reconstructed TIFF image was 16 bits. We applied a line-profile analysis of the voxel values to the 16-bit image exhibiting beam hardening to evaluate the effects of the reconstruction filters on the degree of cupping artifact. The ImageJ program developed at the National Institutes of Health was used for the line profile analysis of the 16-bit TIFF image.

The exact solutions corresponding to the KI phantom of *R* = 0.9 cm were calculated as follows. The upper limit of *n* (i.e., *N*), was taken to be 100. Using the X-ray source and LAC spectra in Fig. 2a, values for *μ_n_*(0) were calculated via Equation (A9) for *n* = 1 to 100. These values were subsequently converted to *ν_n_* using Equation (11), and *C_*n*_* was calculated by Equation (10). Finally, *F_*n*_* was determined by Equation (5) to obtain the cupping profile, *f*(*r*).

### 3.2 Search for the best contrast agent

Substance with a high atomic number (e.g., iodine and lanthanoids) is used as a contrast agent in X-ray CT [6]. The use of such heavy elements yields the undesirable cupping artifact [9], which makes the quantitative CT image analysis difficult. The degree of the cupping depends on the atomic number of the heavy element [10]. In this section, we applied the exact solutions obtained in Sec. 2 to the search for the atomic number of the best contrast agent that induces the least cupping artifact.

The degree of the cupping artifact of a cylindrical phantom can be expressed as the difference in the *f* (*r*) values between the center and the rim of the reconstructed phantom image. As for the *f* (*r*) value at the rim, the limiting behavior of Equation (3) described in Appendix 1 ensures the approximation that *f* (*r*) ≈*C*_1_ in the immediate vicinity of the sample rim. Thus, we take that the best contrast agent yields a minimum with respect to the *C*_1_ value for a fixed *f* (0) value.

We consider the following simple case. A mixture of simple substance of a heavy element and pure water is supposed as a cylindrical homogeneous phantom. The mixture is an aqueous solution or a fine-grained suspension, so that the grain size of the simple substance is very small compared with the voxel dimension, and the mixture can be well approximated as homogeneous.

Heavy elements ranging from _49_In to _79_Au were examined. The *f*(0) value was tentatively fixed to be 0.48 1/cm (≈ 1000 Hounsfield unit (HU)) in the present study. The detailed input data for the exact solutions were as follows. The radius of the phantom, *R*, was 0.9 cm, *N*= 100, and the X-ray source spectrum was for the 100 kV acceleration, which was the same as the simulation in Sec. 3.1. We obeyed Nakashima and Nakano [10, 20] for the estimation of the bulk density of the mixture, which was needed for the LAC spectrum calculation. An example for the calculated LAC spectrum is shown in Fig. 2a for the samarium-water mixture system.

## 4 Results

### 4.1 Effects of reconstruction filters

The synthetic 2-D CT image of the cylindrical phantom of the 440 mM KI solution is shown in Fig. 2b. The original 512^2^ voxels image was trimmed to 300^2^ voxels to provide an expanded view of the cylindrical sample. This figure demonstrates a characteristic beam hardening artifact (cupping phenomenon) along with a non-uniform distribution of the reconstructed LAC values within the sample (see the bright voxels near the sample rim). The line profile along the radial direction (the yellow baseline in Fig. 2b) is provided in Fig. 2c, and also shows the cupping phenomenon. Figure 2c is enlarged in Fig. 2d to enhance the differences between the three reconstruction filters.

With regard to the analytical solutions, the theoretical cupping profile, *f*(*r*), calculated using Equations (3) and (4) is presented in Fig. 2 c and d. A data point spacing of 0.001 cm was applied in the case of this theoretical plot, which is an order of magnitude smaller than the detector spacing (i.e., & *δ* = 0.01 cm) used for the simulation. This was done because the theoretical profile is for an infinite Nyquist wavenumber (an infinitesimal & *δ*). The *μ_n_*(0), *ν_n_*, *C_*n*_*, and *F_*n*_* values calculated for Fig. 2 are summarized in Table 1 for the *n* values up to ten. The two non-linear projections (*p*(*s*), directly calculated by Equations (1) and (7), and *h*(*s*), indirectly calculated by Equation (2) via *μ_n_*(0)) are plotted in Fig. 2e, and demonstrate reasonable agreement.

**Table 1:** List of the coefficients, *μ_*n*_*(0), *ν_*n*_*, *C_*n*_*, and *F_*n*_* in Fig. 2 for the *n* values up to ten. The units of these four coefficients are common, but depend on *n* (i.e., 1/cm*^*n*^*). The two values for *n* = 0 (i.e., *μ*_0_(0) = 1 and *ν*_0_ = 1) are dimensionless constants that are independent of the size and chemistry of the sample

*n*	*μ_*n*_*(0)	*ν_*n*_*	*C_*n*_*	*F_*n*_*
0	1	1	N/A	N/A
1	0.96208	−0.96208	0.96208	0.96208
2	1.14125	0.57062	−0.10783	−0.27458
3	1.60713	−0.26786	0.01570	0.09421
4	2.56714	0.10696	−0.00045	−0.00605
5	4.47574	−0.03730	−0.00056	−0.01666
6	8.28798	0.01151	0.00014	0.00920
7	16.01007	−0.00318	2.4 e-7	3.4 e-5
8	31.88811	0.00079	−8.3 e-6	−0.00247
9	64.98430	−0.00018	1.8 e-6	0.00113
10	134.79017	3.7 e-5	1.2 e-7	0.00016

**Fig. 3:**
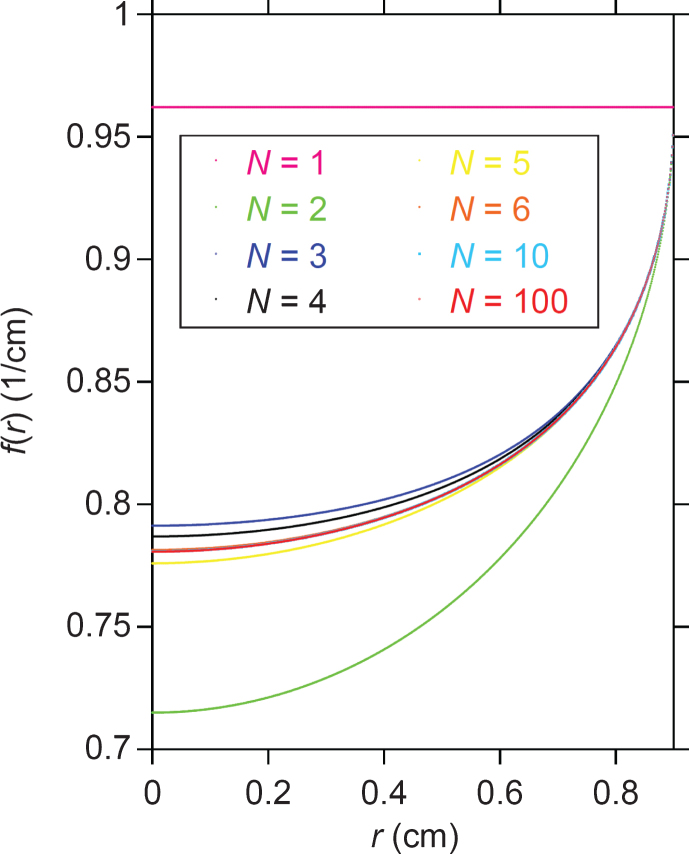
The dependence of the exact solution, *f*(*r*), on *N*. Here, *f*(*r*) for *N* = 100 is the same as that plotted in Fig. 2c, and it is difficult to distinguish the *f*(*r*) curves for *N* = 6, 10, and 100 due to the good agreement.

The effects of the *N* value in Equation (3) for the case of Fig. 2 are shown in Fig. 3. The *f*(*r*) value is constant (i.e., *f*(*r*) = *C*_1_ = *μ*_1_(0)) and no beam hardening occurs if *N* = 1, which is a consequence of assuming that the projection, *h*(*s*), in Equation (2) is linear with respect to *s*. From this figure it is evident that Equation (3) converges rapidly on a fixed profile in conjunction with a small degree of oscillation as *N* increases.

### 4.2 Search for the best contrast agent

**Fig. 4:**
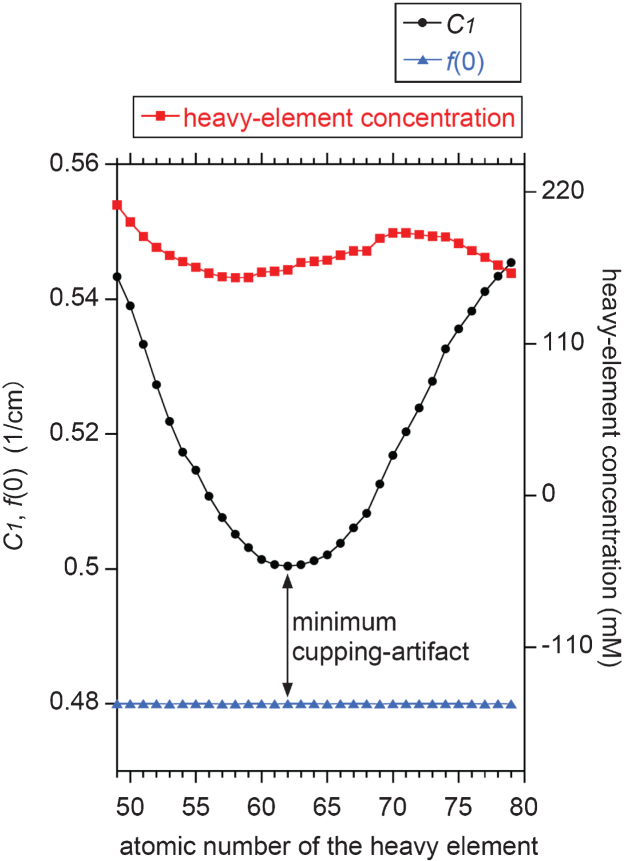
Dependence of *C*_1_ value on the atomic number of the heavy element suspended in the water sample for a fixed *f* (0) value of 0.48 1/cm. The corresponding molar concentration of the heavy element is also plotted. Note that samarium (atomic number, 62) yields the minimum cupping artifact of *C*_1_ – *f* (0) ≈ 0.02 1/cm, for which the corresponding LAC spectrum is shown in Fig. 2a.

Using the X-ray source spectrum in Fig. 2a and a LAC spectrum of a mixture of a specific heavy element and water, values for *μ_n_*(0) were calculated via Equation (A9) for *n* = 1 to 100. These values were subsequently converted to *ν_n_* using Equation (11), and *C*_*n*_ was calculated by Equation (10). Finally, *F*_*n*_ was determined by Equation (5) to obtain the cupping profile, *f* (*r*). By trial and error, we searched for the molar concentration of each heavy element in the aqueous solution/suspension which yields *f* (0)= 0.48 1/cm (≈ 1000 HU), and plotted the *C*_1_ value against the atomic number of the heavy element as well as the corresponding molar concentration. The results are summarized in Fig. 4, depicting that the *C*_1_ value is sensitive to the atomic number of the heavy element in the mixture. For example, *C*_1_= 0.522 (≈ 1180 HU), 0.500 (≈ 1080 HU), and 0.545 (≈ 1270 HU) for _53_I, _62_Sm, and _79_Au, respectively. This is a consequence of that the degree of the cupping artifact is sensitive to the relative position between the K absorption edge of the heavy element and the spectrum peak of the X-ray source [9, 10, 20].

## 5 Discussion

From Fig. 2c, it can be seen that the line profile of each simulated image closely coincides with the exact solution. This result confirms that the power-series approximation for the non-linear projection (Equation 2) is reasonable. This is supported by Fig. 2e, depicting good agreement between *h*(*s*) and *p*(*s*). As for the choice of the upper limit of the *n* value (i.e., *N*), a larger *N* value theoretically ensures better accuracy as far as the numerical calculation error is not serious. However, the three profiles for *N* = 6, 10, and 100 yield almost the same curves in Fig. 3, implying that an *N* value as small as approximately 6 would be practically sufficient for the KI-solution sample of Fig. 2. This is also confirmed by the rapid decrease of the *F_*n*_* values with increasing *n* in Table 1.

Figure 2d shows a discrepancy in the line profiles of the simulated images and the exact solution near the sample rim (i.e., at *r* = 0.9 cm). This is attributed to the use of a finite Nyquist wavenumber when generating the simulated image [4, 16]. The discrepancy relative to the exact solution is greatest for the Chesler filter and smallest for the Ramachandran filter. The Chesler and Ramachandran filters are the strongest and weakest low-pass filters among the three options, respectively, and this is reflected in the degree of discrepancy in Fig. 2d. However, the three simulated image line profiles are in reasonable agreement with the exact solution, with the exception of a few voxels at the sample rim (Fig. 2c and d). Thus, we conclude that the exact solution is applicable to the analysis of real-world CT images using either Chesler, Shepp, or Ramachandran filters with finite Nyquist wavenumbers, except in the vicinity of the sample rim.

Figure 4 depicts that the best contrast agent is not conventionally used iodine [9] but samarium [6] because the difference of the *f* (*r*) values between the center and the rim is as small as 0.020 1/cm (≈ 80 HU) for samarium while it is 0.042 1/cm (≈ 180 HU) for iodine. This is a consequence of that the K absorption edge of samarium (47 keV) is located near the spectrum peak of the continuous X-ray component of the source spectrum (see Fig. 2a) [9, 10, 20]. It should be noted that Fig. 4 was obtained not by the conventional image-reconstruction numerical simulations having undesirable errors [4, 10, 37] but by the analytical solutions. The analytical solutions derived in the present study would contribute to the reliable assessment of the contrast agent performance without such undesirable errors.

As can be seen from Equations (11), (10), and (5), the *n*th moment of the LAC (i.e., *μ_n_*(0)) and the non-linearity of the projection (i.e., *C_*n*_*) are mathematically related to the coefficient, *F_*n*_*, for the line profile, *f*(*r*). This set of analytical expressions can be considered as either a forward or inverse problem. Assuming a forward problem [4, 10, 20], it is possible to predict the line profile, *f*(*r*), for the reconstructed CT image of a sample with a known size and chemistry if the X-ray source spectrum or thickness-dependent projection data are available. This would have applications in assessing the performance of a CT imaging system using a cylindrical phantom as demonstrated in Fig. 4. In the case of an inverse problem [18, 21, 22], the same set of analytical expressions allows us to estimate the X-ray source spectrum by analyzing the line profile data, *f*(*r*), experimentally obtained using cylindrical phantoms of known size and chemistry. In term of the mathematical inverse problem, the use of plural phantoms with different heavy elements is preferable because it would increase the estimation accuracy of the X-ray source spectrum. For example, the *C*_1_ values for _58_Ce and _74_W are significantly different in Fig. 4, and both can be independent constraint in solving the inverse problem. Thus, the simultaneous use of the _58_Ce- [20] and _74_W-water [9] phantoms would contribute to the more accurate estimation of the source spectrum.

The exact solutions obtained are applicable not only to X-ray CT [13, 23–26, 34], but also to other diverse imaging systems employing straight ray paths. Example includes those employing neutrons, positrons, muons, and neutrinos [27–30]. The analytical expressions for a homogeneous cylindrical phantom could also be used to assess imaging systems for a wide range of CT applications.

## 6 Conclusions

Analytical expressions were obtained for the reconstructed image of a homogeneous cylindrical sample exhibiting beam hardening (or cupping artifact) due to a polychromatic X-ray source. The quantitative relationship between the cupping profile and the LAC and X-ray source spectra was derived from non-linear projection data with respect to the penetration thickness by employing Ramachandran filtering with an infinite Nyquist wavenumber. The cupping profile resulting from the analytical expressions was in reasonable agreement with that generated by numerically simulated CT imagery in conjunction with various reconstruction filters (Ramachandran, Shepp, and Chesler filters with a finite Nyquist wavenumber), except for a few voxels near the sample rim. Application of the analytical expressions to the best choice of the contrast agent that induced the least cupping artifact was also demonstrated successfully. The exact solutions as determined in the present study are expected to have applications in the analysis of cylindrical phantom data during the evaluation of real-world CT systems using such practical reconstruction filters.

## Conflict of interest

The authors declare that they have no conflict of interests.

## Appendix 1: Filtered backprojection for a homogeneous cylindrical sample

The derivation of Equations (3) and (4) from Equation (2) using the conventional filtered backprojection method [4, 16, 17] is described herein. First the one-dimensional Fourier transform of the projection was calculated, second the Ramachandran filtering followed by the inverse Fourier transform was performed, and finally the backprojection operation was employed for the reconstruction of the one-dimensional image, *f*(*r*).

Applying a one-dimensional Fourier transform to the projection *h*(*s*), the quantity *P*(*z*) is obtained as:

(A1) $$\begin{array}{c} P(z)\;=\;\int_{-\infty }^{\infty }h(s)\exp\left(-2\pi \mathrm{izt}\right)\mathrm{dt}\\ =\;2\int_{0}^{R}h(2\sqrt{R^{2}-t^{2}})\cos(2\pi \mathrm{zt})\mathrm{dt}, \end{array}$$

where *z* is the wavenumber and the parameter *t* (= *r*sin*θ*) is as indicated in Fig. 1. The evenness of *h* with respect to *t* is also incorporated into the above equation.

Equation (2) was substituted into Equation (A1) and the integral formula (ET I 11(8)) of [31] was used to obtain:

(A2) $$\begin{array}{c} P(z)\;=\;\sum_{n=1}^{N}C_{n}2^{n+1}\int_{0}^{R}{\left(R^{2}-t^{2}\right)}^{\frac{n}{2}}\cos(2\pi \mathrm{zt})\mathrm{dt}\\ =\;\sum_{n=1}^{N}C_{n}{\left(\frac{2}{\sqrt{\pi }}\right)}^{n}{\left(\frac{R}{z}\right)}^{\frac{n+1}{2}}\Gamma \left(\frac{n}{2}+1\right)J_{\frac{n+1}{2}}\left(2\pi \mathrm{Rz}\right), \end{array}$$

where *J*_(*n*+1)/2_ (2*πRz*) is the Bessel function of the first kind.

Ramachandran filtering (i.e., |*z*|) with an infinite Nyquist wavenumber was applied to *P*(*z*) to obtain the quantity *q*(*t*), written as:

(A3) $$\begin{array}{c} q(t)\;=\;\int_{-\infty }^{\infty }P(z)\left|z\right|\exp\left(2\pi \mathrm{itz}\right)\mathrm{dz}\\ =\;2\int_{0}^{\infty }P(z)z\cos(2\pi \mathrm{tz})\mathrm{dz}. \end{array}$$

The evenness of the integrand was incorporated into the above equation.

As a final step, the backprojection calculation was applied to Equation (A3) to obtain *f*(*r*), as:

(A4) $$\begin{array}{c} f(r)\;=\;\int_{0}^{\pi }q\left(r\sin\theta \right)d\theta \\ =\;2\int_{0}^{\infty }\mathrm{zP}\left(z\right)\int_{0}^{\pi }\cos\left(2\pi \mathrm{zr}\sin\theta \right)d\theta \mathrm{dz}\\ =\;2\pi \int_{0}^{\infty }\mathrm{zP}\left(z\right)J_{0}\left(2\pi \mathrm{rz}\right)\mathrm{dz}. \end{array}$$

The Bessel’s integral formula for *J*_0_ (2*πrz*) [32] was used in the conversion. The substitution of Equation (A2) into Equation (A4) then yields:

(A5) $$f(r)\;=\;2\pi \sum_{n=1}^{N}C_{n}{\left(\frac{2}{\sqrt{\pi }}\right)}^{n}R^{\frac{n+1}{2}}\Gamma \left(\frac{n}{2}+1\right)\int_{0}^{\infty }z^{\frac{1-n}{2}}J_{\frac{n+1}{2}}\left(2\pi \mathrm{Rz}\right)J_{0}\left(2\pi \mathrm{rz}\right)\mathrm{dz}.$$

In the case that 0 ≤ *r* < *R*, the integral formula (11.4.41) of [33] can be applied to Equation (A5) to obtain Equation (3). However, at *r* = *R*, different formulae were used as follows. According to the formula (6.574) of [31], all the terms for *n*≥2 on the right-hand side of Equation (A5) is zero. Only the term for *n* = 1 survives in Equation (A5), and the formula (11.4.42) of [33] was applied to the term to obtain Equation (4).

According to Equation (3) for 0≤*r* < *R*, the limiting behavior at the sample rim is that *f* (*r*) ⟶ *C*_1_ as *r* ⟶*R*. Thus, it should be noted that *f*(*r*) ≈*C*_1_ in the immediate vicinity of the sample rim, and is therefore independent of the *N* value (see Fig. 3) and the sample size (*R*). Because *f*(*R*) =*C*_1_/2 (see Equation (4) and Fig. 2d), there is a gap in the *f* (*r*) values between *f* (*r*) as *r* ⟶*R* and *f* (*R*), which often occurs in the case of a Fourier transform involving a discontinuity.

The following equations may be helpful to the practical numerical calculation. We introduce the coefficient *G_*n*_* that is independent of the sample chemistry and the X-ray source spectrum as defined by:

(A6) $$G_{n}\;=\;\frac{2}{\sqrt{\pi }}\frac{\Gamma \left(\frac{n}{2}+1\right)}{\Gamma \left(\frac{n+1}{2}\right)}\;\;\;\;\;\;\;\;\;\;\mathrm{for}\;n\;=\;1,\;2,\;3,\;\ldots ,\;N,$$

to rewrite Equation (3) as:

(A7) $$f(r)\;=\;\sum_{n=1}^{N}{2^{n-1}G_{n}C_{n}\left(R^{2}-r^{2}\right)}^{\frac{n-1}{2}}\;\;\;\;\;\;\;\;\;\;\;\mathrm{for}\;0\;\leq \;r\;<\;R.$$

The ratio of the gamma functions in Equation (A6) can be readily simplified to obtain:

$$G_{n}\;=\;\frac{2}{\pi }\frac{n}{G_{n-1}},$$

and

(A8) $$G_{0}=\frac{2}{\pi }\;\;\;\;\;\;\;\;\;\mathrm{for}\;n\;=\;1,\;2,\;3,\;\ldots ,\;N.$$

The use of the compact recurrence relation, Equation (A8), may be helpful to the effective computer programing for the calculation of *f*(*r*).

## Appendix 2: The relationship between the non-linear projection and the *n*th moment of the LAC spectrum

The derivation of Equation (10) from Equations (8) and (9) is described herein. First, we derive recurrence relations on the *n*th moment by differentiation with respect to *s*. Second, we combine the recurrence relations with Equation (2) to relate *μ_n_*(0) to *C_n_*. Starting from Equations (7) to (9), we can derive the following set of equations:

$$M_{0}(s)\;=\;I(s),$$

$$\frac{{\mathrm{dM}}_{n}(s)}{\mathrm{ds}}\;=\;-M_{n+1}(s)\;\;\;\;\;\;\;\mathrm{for}\;n\;=\;0,\;1,\;2,\;\ldots ,\;N-1,$$

$$\frac{{\mathrm{dM}}_{0}(s)}{\mathrm{ds}}\;=\;\frac{\mathrm{dI}}{\mathrm{ds}}\;=\;-M_{1}(s),$$

$$\mu_{n}(0)\;=\;\int_{0}^{\infty }i_{0}\left(E\right)\mu (E{)}^{n}\mathrm{dE}\;\;\;\;\;\;\;\mathrm{for}\;n\;=\;0,\;1,\;2,\;\ldots ,\;N,$$

$$\mu_{0}(s)\;=\;1,$$

and

(A9) $$\begin{array}{ccc} \frac{d\mu_{n}}{\mathrm{ds}}\;=\;\frac{1}{I^{2}}\left(\frac{{\mathrm{dM}}_{n}}{\mathrm{ds}}I-M_{n}\frac{\mathrm{dI}}{\mathrm{ds}}\right)\;=\;\frac{-M_{n+1}I+M_{n}M_{1}}{I^{2}}\;=\;\mu_{1}\mu_{n}-\mu_{n+1} & & \\ \mathrm{for}\;n\;=\;0,\;1,\;2,\;\ldots ,\;N-1. & & \end{array}$$

Using the expressions in Equation (A9) with respect to *μ_n_*, we obtain:

(A10) $$\mu_{n+1}\;=\;\sum_{m=0}^{n}{\left(-1\right)}^{m}\left(\begin{array}{c} n\\ m \end{array}\right)\mu_{n-m}\frac{d^{m}\mu_{1}}{ds^{m}}\;\;\;\;\text{for}\;n\;=\;1,\;2,\;3,\;\ldots ,N-1,$$

where

(A11) $$\frac{d^{0}\mu_{1}}{{\mathrm{ds}}^{0}}\;=\;\mu_{1},$$

and

(A12) $$\left(\begin{array}{c} n\\ m \end{array}\right)\;=\;\frac{n!}{m!(n-m)!}.$$

Equation (A10) can be rewritten as:

(A13) $$\frac{d^{n}\mu_{1}}{{\mathrm{ds}}^{n}}\;=\;{\left(-1\right)}^{n}\mu_{n+1}-\sum_{m=0}^{n-1}{\left(-1\right)}^{n-m}\left(\begin{array}{c} n\\ m \end{array}\right)\mu_{n-m}\frac{d^{m}\mu_{1}}{{\mathrm{ds}}^{m}}\;\;\;\;\;\mathrm{for}\;n\;=\;1,\;2,\;3,\;\ldots ,\;N-1.$$

The moment described can be related to the power-series projection, *h*(*s*). Based on Equations (1), (9), and (A9), the first derivative of *h*(*s*) is expressed as:

(A14) $$\frac{\mathrm{dh}}{\mathrm{ds}}=\frac{\mathrm{dI}}{\mathrm{ds}}\frac{d}{\mathrm{dI}}\left(-\ln I\right)\;=\;\frac{M_{1}}{I}\;=\;\mu_{1}.$$

The substitution of Equation (2) into Equation (A14) then yields:

(A15) $${\frac{d^{n}h}{{\mathrm{ds}}^{n}}|}_{s=0}\;=\;{\frac{d^{n-1}\mu_{1}}{{\mathrm{ds}}^{n-1}}|}_{s=0}\;=\;n!C_{n}\;\;\;\;\;\;\;\;\;\;\;\;\mathrm{for}\;n\;=\;1,\;2,\;3,\;\ldots ,\;N.$$

As a final step, Equation (A15) is substituted into Equation (A13) for *s* = 0 to relate *μ_n_*(0) to *C_*n*_* using the following recurrence relation:

(A16) $$\begin{array}{c} C_{n+1}\;=\;-\left[\frac{{\left(-1\right)}^{n+1}\mu_{n+1}(0)}{\left(n+1\right)!}+\frac{1}{\left(n+1\right)!}\sum_{m=0}^{n-1}\frac{{\left(-1\right)}^{n-m}n!\left(m+1\right)!\mu_{n-m}(0)}{m!\left(n-m\right)!}C_{m+1}\right]\\ =\;-\left[\frac{{\left(-1\right)}^{n+1}\mu_{n+1}(0)}{\left(n+1\right)!}+\sum_{m=0}^{n-1}\frac{{\left(-1\right)}^{n-m}\mu_{n-m}(0)}{\left(n-m\right)!}\frac{m+1}{n+1}C_{m+1}\right]\\ \;\;\;\;\;\;\;\;\mathrm{for}\;n\;=\;0,\;1,\;2,\;3,\;\ldots ,\;N-1. \end{array}$$

Equation (A16) can be simplified to Equation (10) using *ν_n_* as defined by Equation (11). It should be noted that if *n* = 0, according to the conventional rule, the sum operation of the second term of the right-hand side of Equations (10) and (A16) is not performed and only the first term is calculated.

It may be helpful to explicitly show *C_n_* for the first three values. These are:

$$C_{1}\;=\;\mu_{1}(0),$$

$$C_{2}\;=\;\frac{\mu_{1}(0{)}^{2}-\mu_{2}(0)}{2},$$

and

(A17) $$C_{3}\;=\;\frac{2\mu_{1}(0{)}^{3}-3\mu_{1}(0)\mu_{2}(0)+\mu_{3}(0)}{6}.$$

The following simple relationship between the normalized X-ray intensity after sample penetration (i.e., *I*(*s*)/*I*(0)) and *ν_n_* should be noted:

(A18) $$\frac{I(s)}{I(0)}\;=\;\int_{0}^{\infty }i_{0}\left(E\right)\exp\left(-\mu (E)s\right)\mathrm{dE}\;=\;\int_{0}^{\infty }i_{0}\left(E\right)\sum_{n=0}^{\infty }\frac{{\left[-\mu (E)s\right]}^{n}}{n!}\mathrm{dE}\;=\;\sum_{n=0}^{\infty }\nu_{n}s^{n}.$$

The power-series (i.e., Taylor-series) expansion of the natural exponential function was employed in the derivation of Equation (A18).
